# Unintentional injuries among children aged 1–5 years: understanding the burden, risk factors and severity in urban slums of southern India

**DOI:** 10.1186/s40621-018-0170-y

**Published:** 2018-11-05

**Authors:** Srujan Lam Sharma, Samarasimha Reddy N, Karthikeyan Ramanujam, Mats Steffi Jennifer, Annai Gunasekaran, Anuradha Rose, Sushil Mathew John, Anuradha Bose, Venkata Raghava Mohan

**Affiliations:** 1 Department of General Surgery, Christian Medical College, Vellore, Tamil Nadu 632004 India; 2 The Wellcome Trust Research Laboratory, Division of Gastrointestinal sciences, Christian Medical College, Vellore, Tamil Nadu 632004 India; 3 Department of Community Health, Christian Medical College, Vellore, Tamil Nadu 632004 India; 4 Low Cost Effective Care Unit, Christian Medical College, Vellore, Tamil Nadu 632001 India

**Keywords:** Unintentional injuries, Environmental hazards, Hazard score, Under-five children, Urban slums, India

## Abstract

**Background:**

Globally, 5.82 million deaths occurred among children under the age of five years in 2015 and injury specific mortality rate was 73 per 100,000 population. In India, injury specific mortality rate is around 2.1 per 1000 live births contributing to 4% of the total under 5 mortality rate. This study aims to estimate the burden and understand factors associated with unintentional injuries among children aged 1–5 years residing in urban slums of Vellore, southern India. We also attempted to assess the hazards posed by the living environment of these children and study their association with unintentional injury patterns.

**Methods:**

This cross-sectional study was conducted in eight urban slums of Vellore, southern India and primary caregivers of children aged 1–5 years were interviewed with a questionnaire to obtain the details of injuries sustained in the past three months. Environmental hazard risk assessment was conducted at places frequented by these children and their scores calculated. Baseline prevalence and incidence rates of unintentional injuries were estimated. Multivariate logistic regression and poisson regression analysis were performed to examine factors associated with unintentional injuries and repeated injuries respectively. Association between environmental hazard risk and unintentional injuries was estimated.

**Results:**

Prevalence of unintentional injuries was 39.1% (95% CI 35.4–42.9%) and incidence rate was 16.5 (95% CI 14.7–18.3) per 100 child months (*N* = 662). Bivariate analysis revealed that children of working mothers (OR 1.48; 1.01–2.18) and children from overcrowded families (OR 1.78; 1.22–2.60) had increased odds of sustaining unintentional injuries. Multivariate regression analysis revealed that children from overcrowded families had increased odds of sustaining unintentional injuries (AOR 1.66, 95% CI 1.14–2.41). Boys (IRR 1.33, 95% CI 1.07–1.66) and children from overcrowded families (IRR 1.50; 1.14–1.98) were at increased risk of having repeated injuries. There is an increase in incidence rate of injuries with an increased environmental hazard risk, although not statistically significant.

**Conclusions:**

The burden of unintentional injuries was very high among study children when compared to studies in other urban slums in India. Environment plays an important role in the epidemiology of unintentional injuries; providing safe play environment and adequate supervision of children is important to reduce its burden.

**Electronic supplementary material:**

The online version of this article (10.1186/s40621-018-0170-y) contains supplementary material, which is available to authorized users.

## Background

Globally, 5.82 million deaths occurred among children under the age of five years in 2015. (Global Burden of Disease Child and Adolescent Health Collaboration et al. 2017). The injury specific mortality rate in the under five age group was 73 per 100,000 population and 3654 years of life were lost per 100,000 population (WHO 2015). Among children aged 1–5 years, injuries are the leading cause of death in the developed world (Sminkey 2008). Additionally, there is an unequal distribution between the developed and the developing world, with the mortality rate from unintentional injuries in developing countries being nearly twice that of the developed world (Chandran et al. 2010). According to the World Health Organization (WHO), up to 50% of the children presenting to a hospital with unintentional injuries are left with some form of disability (Peden et al. 2008). More than 95% of all the injury deaths in children occur in the Low and Middle Income countries; children in Southeast Asia have the second highest rates (49/100,000) of unintentional injuries in the world (Peden et al. 2008).

According to World Health Statistics 2015, overall under 5 mortality rate (U5MR) in India in 2013 is 52.7 per 1000 live births and injury specific mortality rate is around 2.1 per 1000 live births contributing to 4% of the total U5MR (WHO 2015). In a national survey based on verbal autopsy, the mortality rate related to injuries among children under 5 years was 302 per 100,000 live births (Jagnoor et al. 2011). Studies from rural Andhra Pradesh and Tamil Nadu have documented injury rates of 307 and 342 per 1000 child-years respectively (Nirgude et al. 2012; Sivamani et al. 2009). Unintentional injuries thus lead to substantial morbidity and mortality in children younger than five years of age in India. Children under the age of one have different patterns of injuries with most injury-related deaths attributed to suffocation as a result of an unsafe sleeping environment (Borse et al. 2008; Imamura et al. 2012). At the age of 1–5 years, children start to move more independently and this increases their risk of injury. A multinational study conducted in developing nations found that children aged 1–5 years sustain injuries with the most long-term consequences with high mortality rates (Hyder et al. 2009; Morrongiello and Matheis 2007).

In India, unplanned urbanization and rise in informal settlements are leading to an increase in urban slums thus leading to high population density and overcrowding (Bandyopadhyay and Agrawal 2013; Tripathi 2015). Lack of spaces in Indian towns and cities due to rapid urbanization has increased the environmental hazards and as a consequence, injuries of all types including unintentional injuries among younger age groups have increased (Naeini et al. 2011; Nambiar et al. 2017). In India, since most under five deaths still continue to be due to infectious causes, more emphasis is placed on vaccine-preventable diseases and there is a lack of policy focus and planning directed at unintentional injuries, a preventable source of significant morbidity and mortality (Fadel et al. 2017). With the paucity of literature regarding childhood unintentional injuries in India, this study aims to estimate the burden and examine factors associated with unintentional injuries among children aged 1–5 years residing in urban slums of Vellore city in Tamil Nadu, southern India. We have also attempted to assess the hazards posed by the living environment of these children and study their association with unintentional injury patterns.

## Methods

This cross-sectional study was conducted between February to October 2013 in eight urban slums of Vellore city, Tamil Nadu, southern India and the period of data collection was from April through August 2013. The urban slums included Old town, Salavanpet, and neighboring areas with a population of around 13,000 with an average family size of 5.7 (3–13), population density of 42,000 per km^2^. Previous studies from similar urban slums in Vellore have reported an infant mortality rate of 38 per 1000 live births (John et al. 2014). Children between one and five years of age, with mothers being the primary caregiver and families residing in the study area for more than three months preceding the study were included. In case of more than one eligible child in the household, the youngest child was included. Children with primary caregivers other than mothers were excluded as previous studies show an association between children’s risky behaviour, injury rate and mother’s locus of control (Damashek et al. 2005). Assuming 50% injury prevalence in the one to five years’ age group and 10% relative precision at 95% confidence level, the calculated sample size was 384 (Hajian-Tilaki 2011; McGee et al. 2004). A door-to-door survey was conducted in the eight urban slums that identified 662 children in the study area who were satisfying our inclusion criteria and consenting to participate in the study. We decided to include all of the eligible children as a larger sample size would facilitate the evaluation of the Environmental Hazard Score (EHS). The study investigators (SLS and MSJ) have trained the field research assistants to administer the questionnaire, which was piloted in an urban slum that did not participate in the study and was modified based on feedback from piloting. Each primary caregiver was briefed about the study and trained field research assistants obtained written informed consents before administering the questionnaire (Additional file 1). The interview was held in the subjects’ home at a time convenient for the mother, mostly in the mornings. The questionnaire captured details regarding family demographics and the details of injury, including frequency, type, location, and severity of injuries sustained by the children during a period of three months prior to the date of interview and written in the questionnaire forms; these forms were reviewed by the field supervisors on a daily basis and then sent for data entry. Injuries were classified according to the International Statistical Classification of Diseases and related Health problems 10th revision (ICD-10). The study was approved by Institutional Review Board (IRB) of Christian Medical College, Vellore (8078 dated 21.11.2012).

### Environmental hazard score (EHS)

An indigenously developed Environmental hazard form (EHF) was used to assess the risk of different types of injuries such as falls, burns (thermal, chemical, and electric), road traffic injuries, poisoning, drowning, and mechanical hazards from household objects. For risk assessment, a base score (ranging from 0 to 4) was assigned based on the perceived severity for each type of injury mentioned above. A modifying factor score for each type of injury (0–4) was assigned based on the child’s accessibility to the injury risk. The end hazard score for each injury type was calculated by multiplying the base score and the modifying factor score (For example, the base score for a child falling from roof is 4. The modifying factor score was based on accessibility. Scenario 1: If the child had no access to the roof the modifying score was 0. Hence the overall score was 4 × 0 = 0. Scenario 2: However if the child had access to a roof without a parapet (modifier of 2) the score was 4 × 2 = 8″) (Additional file 2: Table S1). Sum of all the end scores from all the locations assessed gives the environmental hazard score for each individual. Environmental data were collected by the trained field research assistants for each child using the developed EHF at four common locations which included homes, play areas surrounding the home, schools and places where a child spent part of the average day (babysitter’s home). The data were collected at all the four locations or only at locations applicable to each child according to his/her age. Finally, the total environmental hazard score (EHS) for each study child was calculated by taking the average of the hazard scores from all the available locations (Additional file 3). The environmental hazard risk was categorised as low risk (≤33 centile), moderate risk (34–66 centiles) and high risk (> 66 centile).

### Definitions of injuries, outcome, and explanatory variables

The primary outcome of the study was to assess the presence of UI in the children during the past three months from the date of data collection. Unintentional Injuries are defined as injuries occurring in short period of time with an unsought outcome and as a result of one of the forms of physical energy in the environment or normal body functions being blocked by external means (Christoffel et al. 1992). The operational definitions of different types of UI used in this study are as follows: fall as injury due to fall to the ground or fall on the ground; burns as injury that causes burns of any degree to the body tissue; electric burns as burn injury due to electrical and electronic gadgets; drowning as submerging in a body of water; poisoning as consumption of non edible substances, including chemicals; road traffic accidents as injury to any part of the body due to moving automobiles; heavy mechanical injury as injury due to a heavy object. Severe UI were defined as those UI requiring medical attention, that is, those that needed a visit to the hospital or treatment by a physician. Families with more than two persons living per room were defined as overcrowded dwellings. Modified kuppuswamy scale was used for assessing the socioeconomic status (Bairwa et al. 2013). Housing type was considered as “pucca” if houses were made with high quality materials throughout, including the floor, roof, and exterior walls and houses made from mud, thatch, or other low-quality materials were called kutcha houses (Ministry of Statistics and Programme Implementation, GOI 2013).

### Statistical analysis

Double data entry was done using Epi-info software and data analyses were performed using STATA 13 software (StataCorp, College Station, TX, USA). The overall prevalence and incidence rates of UI per 100 child months with 95% confidence intervals were estimated based on injuries over a three month recall period which is three months prior to the date of interview. Bivariate analyses were performed to examine associations between socio-demographic factors and unintentional injuries among children. All socio-demographic factors with *p* < 0.2 on bivariate analyses were included in a multivariable logistic regression model to examine the factors associated with unintentional injuries after adjusting for all the potentially confounding co-variates. Socio-demographic factors associated with repeated unintentional injuries were assessed using a Poisson regression model and over-dispersion was adjusted using a quasi-Poisson procedure. UI burden was estimated for children exposed to varying levels of environmental risk and a linear trend analysis was performed to examine association between environmental hazard risk categories and burden of UI.

## Results

A total of 662 children were surveyed of which, 321(48.5%) were boys and 341(51.5%) were girls. Majority, 611(92.3%) belonged to lower socioeconomic group and 356 (53.8%) were Hindus. The median (IQR) family size was 5(4–7) and 361(55%) families had ≤2 children in the house. Predominantly, 499 (75.4%) families had ≤2 rooms in the house and 273 (41.3%) families lived in pucca houses. The mean (SD) years of schooling among mothers was 7.6 (2.8) and 87 (13%) mothers of study children had no formal schooling. The mean (SD) age of mothers was 26.4 (4.2) years and about 22% (146/662) of them were gainfully employed (Table 1).

**Table 1:** Baseline characteristics of study participants (*N* = 662)

Variables	Category	Frequency (n)	Percentage (%)
Gender	Male	321	48.5
	Female	341	51.5
Number of children in the family	≤ 2	361	55.0
	> 2	301	45.0
Number of adults in the family	≤ 2	342	52.0
	> 2	320	48.0
Number of siblings for recruited child	≤ 2	578	87.3
	> 2	84	12.7
Religion	Hindu	356	53.8
	Christian	16	2.4
	Muslim	290	43.8
Maternal age (years)	≤ median 26	361	55.5
	> median 26	301	45.4
Maternal education	No formal education	87	13
	Primary	168	26
	High school	339	51
	Higher secondary/Graduate and higher	68	10
Maternal occupation	Home maker	516	78
	Employed outside home	146	22
Father living with the child	Yes	637	96.2
	No	25	3.8
Paternal age (years)	≤ median 30	360	54.3
	>median 30	302	45.7
Paternal education	Professional degree	4	0.6
	Graduate/Post graduate degree	18	2.6
	Post high school/diploma	6	0.9
	High school certificate	110	16.6
	Middle school certificate	274	41.3
	Primary school certificate	154	23.2
	No education	96	14.5
Paternal Occupation	Professional	5	0.7
	Semi professional	1	0.1
	Clerical job/shop owner/farmer	21	3.1
	Skilled worker	113	17.0
	Semi-skilled worker	96	14.5
	Unskilled worker	392	59.2
	Unemployed	34	5.1
Type of house	Pucca	273	41.3
	Semi pucca	170	25.6
	Kutcha	219	33.1
Family size	≤ 5	354	53.4
	> 5	308	46.6
Number of rooms	≤ 2	499	75.4
	> 2	163	24.6
Reported family income per month (INR)	< 2000	7	1
	2000–5000	416	63
	6000–10,000	224	34
	> 10,000	15	2
Socio-economic status	Lower	2	0.3
	Upper lower	609	92.0
	Lower middle	46	7.0
	Upper middle	5	0.7

### Burden of unintentional injuries

The prevalence of UI among children aged 1–5 years was 39.1% (259/662, 95% CI 35.4–42.9%) over a three-month recall. The prevalence of UI was 42.6% (137/321) among boys and 35.8% (122/341) among girls and this difference was not statistically significant. Proportion of children sustaining UI in the age groups 13–24; 25–36; 37–48 and 49–60 months were 33.7% (95% CI 27.3–40.7); 40.3% (95% CI 33.0–47.5); 40.9% (95% CI 33.5–48.6) and 43.1% (95% CI 35.0–52.0) respectively. Even though the proportion of children getting injured were increasing with age, this association was not statistically significant (*Chi-square trend 2.76, P-value = 0.09*) (Table 2).

**Table 2:** Age specific prevalence and incidence rates of unintentional injuries among study children aged 1–5 years

Age group (Months)	No. of children	No. of injured children	Proportion^a^ (95% CI)	Odds Ratio	No. of Injuries	Incidence rate per 100 child months^b^ (95% CI)
13–24	187	63	33.7 (27.3–40.7)	1	83	14.8 (11.8–18.2)
25–36	186	75	40.3 (33.5–47.5)	1.33 (0.85–2.07)	91	16.3 (13.2–19.9)
37–48	159	65	40.9 (33.5–48.6)	1.36 (0.85–2.16)	81	16.9 (13.6–21.0)
49–60	130	56	43.1 (35.0–52.0)	1.48 (0.91–2.42)	68	17.4 (13.6–21.9)
Overall	662	259	39.1 (35.4–42.9)		323	16.2 (14.6–18.1)

^a^Linear trend analysis for proportion of unintentional injuries with age group (*P* = 0.09, Chi square value = 2.76)

^b^Linear trend analysis for incidence rate with age group (*P* value = 0.743, Chi-square value =1.27)

**Fig. 1:**
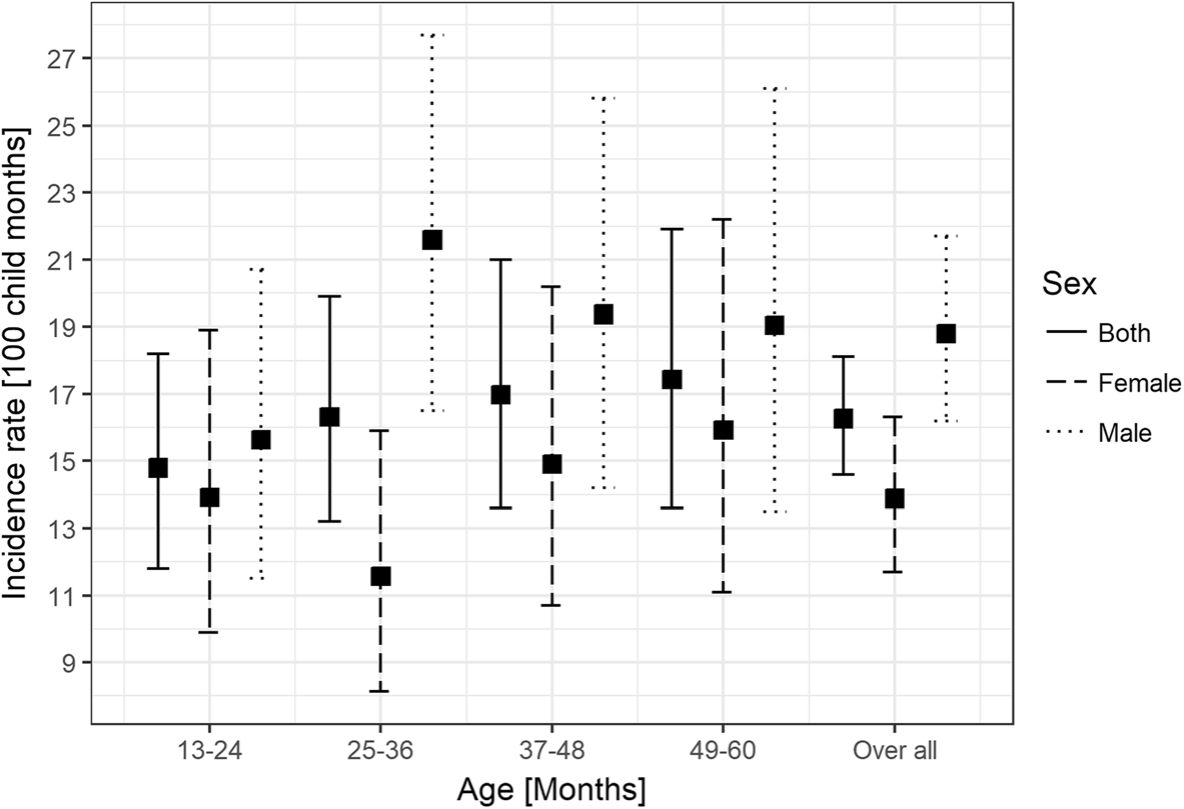
Gender specific incidence rates of unintentional injuries among children of 1–5 years

Incidence rate of UI among the study children aged 1–5 years was 16.2 per 100 child months (95% CI 14.6–18.1). Children in the age group of 4–5 years had a high risk of injury (43.1%, 56/130) with an incidence rate of 17.4 (95% CI 13.6–21.9) per 100 child months (Fig. 1). A total of 323 UI were reported in 259 children with 207 (80%), 42(16.2%) and 10 (3.8%) children experiencing one, two and more than two UI respectively. Among 323 UI, 306 (94.7%) were due to falls, 9 (2.7%) were due to burns/fire injuries and 5 (1.5%) were due to road traffic injuries; injury type could not be determined for three injuries. Out of 306 fall injuries, 171(56%) were among males, 135(44%) were among females and this difference was statistically significant (*P*-value = 0.04). However, there were no statistically significant gender differences in burns and road traffic injuries (Additional file 2: Table S2). Injuries due to drowning, electrocution or poisoning were not reported during the study period.

### Factors associated with unintentional injuries

Bivariate analyses revealed that children of working mothers have significantly higher odds (OR 1.48; 1.01–2.18) of sustaining unintentional injuries than children whose mothers stayed at home. Similarly, children from overcrowded families had significantly higher odds of having an unintentional injury (OR 1.78; 95% CI 1.22–2.60). Other socio-demographic factors including presence of siblings, maternal age and education level; paternal age, education, occupation; family size and income were not significantly associated with higher risk of unintentional injuries (Table 3). Multivariate logistic regression analysis showed that children residing in overcrowded families (AOR 1.66; 95% CI 1.14–2.41) were significantly at higher odds of getting injured after adjusting for gender, presence of siblings, maternal occupation, type of house and overcrowding (Table 3).

**Table 3:** Association between socio-demographic factors and unintentional injuries in the study

Co-variates	Cateogery	Injured in past 3 months	Uninjured in past 3 months	Unadjusted OR^a^ (95% CI)	Adjusted OR^b^ (95% CI)
Gender	Male	137(42.6%)	184(57.4%)	1.34(0.97–1.85)	1.31(0.96–1.81)
	Female	122(35.8%)	219(64.2%)		
Siblings	≤ 2	221(38%)	357(62%)	0.74(0.46–1.22)	1.11(0.69–1.79)
	> 2	38(45%)	46(55%)		
Maternal age	≤ median	145(40.1%)	216(59.9%)	1.10(0.79–1.52)	
	> median	114(37.9%)	187(62.1%)		
Maternal education	0–7 yrs	142(41.3%)	202(58.7%)	1.20(0.87–1.67)	
	≥ 8 yrs	117(36.8%)	201(63.2%)		
Maternal occupation	Home maker	68(46.6%)	78(53.4%)	1.48(1.01–2.18)	1.34(0.92–1.97)
	Employed outside home	191(37.1%)	325(62.9%)		
Father living with the child	Yes	250(39.2%)	387(60.7%)	1.14(0.47–3)	
	No	9(36%)	16(64%)		
Paternal age	≤ median	110(36.4%)	192(63.6%)	0.81(0.58–1.12)	
	> median	149(41.4%)	211(58.6%)		
Paternal Education	0–7 yrs	160(39.7%)	243(60.3%)	1.06(0.73–1.48)	
	≥ 8 yrs	99(38.2%)	160(61.8%)		
Paternal occupation	Unemployed	15(44.1%)	19(55.9%)	1.24(0.57–2.63)	
	working	244(38.8%)	384(61.2%)		
House Type	Others	163(42%)	226(58%)	1.32(0.95–1.85)	1.16(0.83–1.61)
	Pucca	96(35%)	177(65%)		
Family size	≤ 5	137(38.7%)	217(61.3%)	0.96(0.69–1.33)	
	> 5	122(40%)	186(60%)		
Number of rooms	≤ 2	205(41%)	294(59%)	1.40(0.95–2.1)	
	> 2	54(33%)	109(67%)		
Over crowding	> 2 per room	202(43%)	268(57%)	1.78 (1.22–2.6)	1.66(1.14–2.41)
	≤ 2 per room	57(30%)	135(70%)		
Family income per month (INR)	< 4650	137(41%)	198(59%)	1.16(0.84–1.6)	
	4650 or more	122(37.3%)	205(62.7%)		

^a^Bivariate analysis

^b^Multivariable logistic regression analysis

### Factors associated with repeated unintentional injuries

Any child sustaining more than one unintentional injury during the three-month recall period was considered to have repeated UI. Poisson regression analysis for assessing the risk of having repeated UI has revealed that, children from overcrowded families experienced higher risk of repeated unintentional injuries (IRR 1.50; 95% CI 1.14–1.98) after adjusting for age, gender, presence of siblings, maternal occupation, housing type and overcrowding. Boys had significantly increased risk of having repeated injury compared to girls (IRR 1.33, 95% CI 1.07–1.66). Older children (age > 2 years), the presence of siblings, working mothers, and poor housing conditions had higher risk of repeated UI but not at statistically significant level (Table 4).

**Table 4:** Factors associated with repeated unintentional injuries using Poisson regression analysis

Variable	Unadjusted	Adjusted
	IRR	IRR (95% CI)
Age > 2 years	1.11	1.11 (0.87–1.44)
Male	1.35^a^	1.33^a^(1.07-1.66)
Siblings	1.20	1.06 (0.77–1.43)
Mother employed outside home	1.26	1.18 (0.92–1.51)
Type of house (Semi pucca/Kutcha)	1.15	1.04 (0.83–1.31)
Overcrowding > 2 persons/room	1.56^a^	1.50^a^ (1.14–1.98)

^a^ *Statistically significant with a P value < 0.05*

### Environmental hazard risk and association with unintentional injuries

The mean (SD) of total environmental hazard score (EHS) was 24.3(14.5). The highest hazard score was present at babysitter/caregivers home 28.6(12.3) followed by homes of children 28.1(16.8). Play areas had a hazard score of 23.1(13.2) and least hazardous were schools with a score of 18.8(9.7). Based on the 33rd and 66th percentiles of the total EHS, children exposed to areas with scores less than or equal to 18.3 were considered to be at low risk, between 18.4 and 28.5 to be at moderate risk and more than 28.5 to be at high environmental risk for sustaining injuries. Among all the study children, 209(32%) were exposed to low-environmental risk, 218(33.5%) to moderate- and 225(34.5%) children to high environmental risk. The proportions of children sustaining UI were 35.8%, 39.5% and 42.3% in the low, moderate and high environmental risk categories and this rising trend was not statistically significant (Table 5). The incidence rates of UI were 14.8 (95% CI 12.0–18.0), 16.1 (95% CI 13.2–19.6) and 18.6(95% CI 15.6–22.1) among children exposed to low, moderate and high environmental risk. Similarly, the incidence rates of severe UI were 6.4 (95% CI 4.6–8.6), 6.3 (95% CI 4.5–8.4) and 6.5 (95% CI 4.8–8.7) among children exposed to low, moderate and high environmental risk. These increasing rates with increasing environmental exposures were not statistically significant (Table 5).

**Table 5:** Environmental hazard risk and injury rates among the study children

Environmental hazard risk	Number of study children (N)	No of children injured^a^ n (%, 95% CI)	Incidence rate of injuries per 100 child months^b^ (95% CI)	No of children severely injured^c^ n (%, 95% CI)	Incidence rate of severe injuries per 100 child months^d^ (95% CI)
Low (≤ 18.3)	209	75(35.8, 28.4–42.8)	14.8(12.0–18.0)	38(18.2, 13.4–23.8)	6.4(4.6–8.6)
Moderate (18.4–28.5)	218	86(39.5, 32.9–46.3)	16.1(13.25–19.5)	37(17.0, 12.2–22.6)	6.3(4.5–8.4)
High (> 28.5)	225	95(42.3, 35.7–48.9)	18.6(15.6–22.1)	39(17.3, 12.8–22.7)	6.5(4.8–8.7)
Total	652	256(39.3, 35.5–43.1)	16.5(14.7–18.3)	114(17.4, 14.7–20.5)	6.4(5.3–7.6)

^a^Linear trend analysis for number of injured children as per environmental hazard risk cateogeries (*P* = 0.17, Chi square = 1.81)

^b^Linear trend analysis for incidence rate of injury as per environmental hazard risk cateogeries (*P* = 0.18, Chi square value =3.36)

^c^Linear trend analysis for number of severely injured children as per environmental hazard risk cateogeries (*P* = 0.77, Chi square = 0.08)

^d^Linear trend analysis for incidence rate for severe injury as per environmental hazard risk cateogeries (*P* = 0.98, Chi square value = 0.04)

## Discussion

The prevalence of UI among children aged 1–5 years residing in urban slums of Vellore was 39.1% which is much higher compared to 8.5% among children of 0–5 years residing in urban slums of New Delhi (Parmeswaran et al. 2017). Higher injury risk were also seen among the urban slum residents in Chennai and in squatter settlements in Karachi, Pakistan which is now corroborated by our study (Rizvi et al. 2006; Sathiyasekaran 1996). Unintentional injuries in this age group are affected by a combination of various environmental and psychological factors. Particularly in young children, the innate curiosity and desire to experiment are not always matched with the ability to understand or respond to danger. The drive to play can override the need for caution even when an appropriate understanding of danger and ability to respond has been attained by developmental maturity (Bartlett 2002). Additionally, as the child passes through this age group, parents increasingly rely on behavioral modification and their trust in the child’s awareness of environment as the primary preventive measure, as opposed to environmental modification (Morrongiello et al. 2004a). It was also seen that preschoolers over the age of 2.5 years were at higher risk of UI than younger toddlers (Dal Santo et al. 2004).

Overcrowding was a significant risk factor for overall UI rate and also for risk of repeated UI. This finding is worrying considering that urban slums continue to grow and get more crowded every day leading to consequent lack of space in Indian towns and cities (Bandyopadhyay and Agrawal 2013). Socio-demographic factors that affect housing and living conditions in these slums could influence hazard and injury patterns. In our study, we did not find any electrocution related UI but a study from Surat, Gujarat has documented that rates of electrocution from electric sockets were increased among the families of high socioeconomic status, most likely as a result of access to electricity (Chaudhari et al. 2009). The same study also found a relation between precautionary measures and the mothers’ level of education which is a limitation of our study as we did not look for precautionary measures. The UI rates were higher in children with working mothers but there was no increase in the frequency of injuries that required medical attention. Whether there is an actual issue with supervision or if there are psychological factors associated with mothers being forced to leave their children at home and over-reporting injuries compared to their stay-at-home peers remains to be studied in this area. Socio-economic factors have also been shown to affect UI-related death rates in other studies but we did not find any significant relationship between any socioeconomic factors within this community and risk of UI (Hong et al. 2010; Laursen et al. 2008). Demographic factors such as the age of the parents and whether the child lives in a single parent or divorced household, or with both parents have also been implicated as determinants of childhood UI but were not found to be significant in our study (Dudani et al. 2010; Hong et al. 2010).

Consequently, as a step towards identifying the interaction of environmental hazards with these children, a hazard scoring was done not only for each child’s residence but also for their play areas, babysitters’ houses and schools as applicable. For example, play areas that were not cordoned off pose a risk of the child running into traffic, homes that did not have parapets to child accessible roofs posed a higher risk of falls to the ground and open fires or stoves on the ground posed risks of the child getting burned. It has been reported that in a high-risk environment (high EHS), even when the perception of hazard risk is high and maternal supervision is high, the rate of UI remains high (Dal Santo et al. 2004). The higher environmental hazard scores were associated with higher rates of UI and severe UI. Housing other than a pucca house has been shown to influence UI rates and combined with the fact that most UI in young children occur in and around homes it is imperative that necessary interventions be placed in these areas to prevent further injury (Dal Santo et al. 2004; Reading et al. 1999). Behavioral and socio-demographic factors are extremely important in understanding these UI and to attain a ‘holistic’ approach to a possible intervention. This outlines the importance of the identification and elimination of environmental hazards as a first line intervention in reducing the rate of childhood UI; and the urgent need for the same is highlighted by our results showing an increase in incidence rate of UI with an increase in the environmental hazard risk although not at a statistically significant level. Possible reason for not having statistically significant difference of incidence rates with increase of environmental hazard risk could be the smaller sample size. The EHS system designed in this study can be easily used to assess environmental risk across a broader spectrum of areas frequently accessed by children after validating it with a larger sample size.

Parental behavior is another important determinant of UI risk. Although it was beyond the scope of our study, it is important to determine if parents will be willing to implement any intervention and also determine if those interventions will be helpful in reducing the hazards (Dal Santo et al. 2004; Damashek et al. 2005; Dudani et al. 2010; Glik et al. 1991; Hong et al. 2010; Morrongiello et al. 2004b; Russell and Champion 1996). Various factors such as maternal anxiety, maternal stress levels and age of the parents determine childhood injury rates (Hong et al. 2010; Morrongiello et al. 2004b). Mothers who believe that their child getting hurt is not controlled by them, i.e., who have an external locus of control have also been found to have higher UI rates in their children (Damashek et al. 2005). Parental risk perceptions also interact with all the previously outlined factors in determining injury risk and are affected by various socio-cultural factors (Dal Santo et al. 2004; Damashek et al. 2005; Glik et al. 1991; Morrongiello et al. 2004a). For example, having a child recently injured alters the parent’s perception of how dangerous the environment is and this, in turn, may affect the strategies undertaken to prevent further such incidents (Glik et al. 1991; Russell and Champion 1996). A community’s perception of the health problem also has a large role to play in what interventions may be implemented and research in Africa has shown that interventions that are passive, rather than those requiring greater parental effort, are more likely to be welcomed by the community (Larsson et al. 2006; Munro et al. 2006; Ruiz-Casares 2009). Therefore, an appropriate understanding of the parents’ and community’s ideas surrounding childhood unintentional injuries is essential to design any intervention. An interesting new finding in this study is boys had an increased risk of repeated UI. To understand this, further studies on socio-cultural factors and behavioral patterns of children and parents are needed.

It is clear that childhood unintentional injuries are the product of a complex interaction between human behavior (that of the parent and child), the environment they live in and various socio-demographic factors; all these factors are interrelated and influence each other (Glik et al. 1991; Larsson et al. 2006; Munro et al. 2006; Ruiz-Casares 2009; Russell and Champion 1996). However in this study area, with the findings of high rates of UI in a hazard intense environment, it is imperative that provision of safe play environments by reducing the environmental hazards be implemented first and a further behavioral study will only be secondary to the same.

### Limitations

One of the limitations of the study is that children with primary caregivers other than mothers were excluded. Recall bias was a known limitation of the study, which was attempted to overcome by asking how the injury was treated; this allowed mothers to recall the actual event. Interviewer bias was another limitation, although the field research assistants used the same words in each interview and the questionnaire forms crosschecked and verified on a daily basis, they were not auto validated.

## Conclusions

The burden of unintentional injuries was very high among study children when compared to studies in other urban slums. Provision of safe environments and supervision of children is needed to prevent UI considering the high burden in the study population. The indigenously developed environmental hazard score (EHS) could possibly be used as a tool to assess the risk of UI at homes and other places frequently visited by children, after it has been validated with a larger sample size. Recent initiative by the Government of India for building smart cities with planned urbanization should be addressing the environmental hazards posed to the communities. However, policy decisions for community-based interventions with combined engineering, environmental measures need to be implemented to reduce the environmental hazards to children while further research is recommended to ascertain the behavioral and psychological components.

## Additional files

Additional file 1: Questionnaire for childhood injuries (DOCX 21 kb)

Additional file 2: **Table S1.** Example of calculating environmental hazard score for a subject at one location. **Table S2.** Distribution of different types of injuries as per gender (DOCX 14 kb)

Additional file 3: Environmental Hazard Observation Form (DOCX 22 kb)

## Abbreviations

CI: Confidence interval
EHF: Environmental hazard form
EHS: Environmental hazard score
ICD-10-CM: International Classification of Diseases, Tenth Revision, Clinical Modification
IQR: Interquartile range
IRR: Incidence rate ratio
OR: Odds ratio
SD: Standard deviation
SES: Socioeconomic status
UI: Unintentional injuries
WHO: World health organization
