# Supplementary material for: Unintentional injuries among children aged 1–5 years: understanding the burden, risk factors and severity in urban slums of southern India
Source: Inj Epidemiol. 2018 Nov 5;5:41. doi: 10.1186/s40621-018-0170-y (PMC6215788; doi:10.1186/s40621-018-0170-y)
Supplement: Supplementary file 1 — Questionnaire for childhood injuries (DOCX 21 kb) [file 40621_2018_170_MOESM1_ESM.docx]

# QUESTIONNAIRE FOR CHILDHOOD INJURIES

## UID NO:

## FIELD WORKER ID:

## DATE OF INTERVIEW:

## 1. GENERAL DETAILS:

1. Address –

Street -

Locality -

1. Type of house -
2. Number of people residing in the house –
3. Adults
4. Children
5. Number of rooms in the house –
6. Head of household’s relationship to subject –
7. Name and address of school/balwadi the subject is enrolled in –
8. Details for play grounds or relatives’ and/or babysitters’ homes the child spends routine days at; *example in case of mothers working outside the home.*
9. Religion -
10. Caste/Community -

## 2. CHILD DETAILS:

1. Age in months
2. Date of Birth (dd/mm/yy) –
3. Sex –
4. Birth order –
5. Number of living siblings

## 3. MOTHER DETAILS:

1. Age –
2. Occupation –
3. Education –

## 4. FATHER DETAILS:

1. Age –
2. Occupation –
3. Education –
4. Does the father live with the child?

## 5. SOCIOECONOMIC STATUS ASSESSMENT:

Please indicate appropriately

1. EDUCATION:

Professional degree

Graduate/Post graduate degree

Post high school/diploma

High school certificate

Middle school certificate

Primary school certificate

Illiterate

1. OCCUPATION

Profession

*(Ex: doctors, advocates, engineers, chartered accountants, etc.)*

Semi profession

*(Ex: school teachers, class 1 and class 2 officers in Govt. services and companies)*

Clerical job/shop owner/farmer

*(Ex: Clerk, shop owner, businessmen, farm or plantation owner, government servants of class 3 category etc.)*

Skilled worker

*(Ex: electricians, welders, fitters, turners, plumbers, drivers etc.)*

Semi skilled worker

*(Ex: tailors, embroidery workers, weavers etc.)*

Unskilled worker

*(Ex: beedi work, hotel work, construction, mason, fishing, sales, rag picking, street vending, head load work etc.)*

Unemployed

*(Includes retired from any of above)*

1. FAMILY INCOME PER MONTH IN RUPEES – Rs_______________

Also mark the category below

>= 30375

15188 -30374

11362-15187

7594-11361

4556-7593

1521-455

<=1520

## PERCEPTION:

1. What are the hazards your child faces on a routine basis in terms of injuries? – List out for each location your child visits on a average day.
   1. At home/within the courtyard or compound of your house
   2. Around the house
   3. At school
   4. At babysitters’/relatives’ homes if child spends average days there
2. When is the child most prone to get hurt (i.e. during what activities, which time of day, under whose supervision etc)?
3. At which physical location is your child more likely to get injured?
4. What measures have you taken to protect your child from injuries so far; list?
5. Do you think injuries are preventable?

6) If yes to 6.5, then how?

7) If no to 6.5, then why not?

## INJURY RECALL:

1. Was your child injured in the past three months?
2. If yes to 7.1, then how many times?

(For each incident attach INJURY SHEET)

# INJURY SHEET

## UID NO:

## INJURY NO:

## HOW MANY DAYS BEFORE THIS INTERVIEW DID THE INJURY HAPPEN?

## AT WHICH PHYSICAL LOCATION DID THIS HAPPEN?

## DETAILS:

What was/were the injuries in this incident alone – list?

Did the child require medical attention?

If yes for 5.b, then where?

If not for 5.b, then what was done?

Did the injury prevent normal activities?

If yes for 5.e, then for how many days?

Is there any lasting consequence?

If yes to 5.g, then what – list?
