# Supplementary material for: Unintentional injuries among children aged 1–5 years: understanding the burden, risk factors and severity in urban slums of southern India
Source: Inj Epidemiol. 2018 Nov 5;5:41. doi: 10.1186/s40621-018-0170-y (PMC6215788; doi:10.1186/s40621-018-0170-y)
Supplement: Supplementary file 2 — Table S1. Example of calculating environmental hazard score for a subject at one location. Table S2. Distribution of different types of injuries as per gender (DOCX 14 kb) [file 40621_2018_170_MOESM2_ESM.docx]

Table S1: Example of calculating environmental hazard score for a subject at one location

| **Hazard** | **Base score** | **Modifying factor** | **Factor score** | **End score (base * factor)** |
| --- | --- | --- | --- | --- |
| Roofs/ balconies | 4 | 0. Absent/not accessible/high child proof parapet  1. Parapet present – child may fall with some difficulty  2. Parapet absent/ parapet present – child may fall of easily | Scenario 1 – 0  Scenario 2 - 2 | 4 * 0 = 0  4 * 2 = 8 |

Table S2: Distribution of different types of injuries as per gender

| Injury type | Male | Female | Total | p-value |
| --- | --- | --- | --- | --- |
| Falls | 171 (56%) | 135 (44%) | 306 | 0.04^b^ |
| Burns and fire injuries | 4 (44.4%) | 5 (55.6%) | 9 | 0.75 |
| Road traffic injuries | 3(60%) | 2(40%) | 5 | 0.68 |
| Total number of Injuries | 178 (55.6%) | 142 (44.4%) | 320^a^ | 0.04^b^ |

^a^Injury type could not be determined for three injuries

^b^Significance based on statistical test for comparing two rates
