# Supplementary material for: Unintentional injuries among children aged 1–5 years: understanding the burden, risk factors and severity in urban slums of southern India
Source: Inj Epidemiol. 2018 Nov 5;5:41. doi: 10.1186/s40621-018-0170-y (PMC6215788; doi:10.1186/s40621-018-0170-y)
Supplement: Supplementary file 3 — Environmental Hazard Observation Form (DOCX 22 kb) [file 40621_2018_170_MOESM3_ESM.docx]

# Additional file 3: **Environmental Hazard Observation Form**

| **Unique id no** |  |
| --- | --- |
| **Field worker id** |  |
| **Form number** |  |
| **Location** | A separate form must be used for each location. Mark one of the following:   1. The child’s home (including courtyard, if applicable) 2. Play areas surrounding the home 3. Schools 4. Places where child spends part of average day under supervision of caregivers other than mother – babysitter’s home, collective care etc. |

## Scoring tables: For each hazard the highest scoring hazard must be accounted for.

## 1. Fall hazards

| **Hazard** | **Base score** | **Modifying factor** | **Factor score** | **End score (base * factor)** |
| --- | --- | --- | --- | --- |
| 1. Ground | 1 | 0. Even floor/ground  1. Uneven floor/ground  2. Potholes/debris  3. Ditches/pits/small drains child may trip over |  |  |
| 2. Steps/staircases | 3 | 0. Absent/not accessible  1. Parapet present – child may fall with some difficulty  2. Parapet absent/ parapet present – child may fall easily |  |  |
| 3. Debris/ construction material | 2 | 0. Absent/ not accessible  1. Small organized pile in specific area  2. Large/ strewn pile |  |  |
| 4. Elevated platform | 2 | 0. Absent/ not accessible/ no chance of fall  1. Child may reach with some difficulty  2. Easily accessible to the child |  |  |
| 5. Roofs/ balconies | 4 | 0. Absent/not accessible/high child proof parapet  1. Parapet present – child may fall with some difficulty  2. Parapet absent/ parapet present – child may fall of easily |  |  |
| 6. Objects child may fall from – parked vehicles, furniture, etc. | 2 | 0. Absent/ not accessible/ no chance of fall  1. Child might climb with some difficulty  2. Easily accessible to the child |  |  |

## 2. Thermal and chemical burn hazards

| **Hazard** | **Base score** | **Modifying factor** | **Factor score** | **End score** |
| --- | --- | --- | --- | --- |
| 1. Cooking stove/ open fire | 4 | 0. Absent/inaccessible to child  1. Child may reach with some difficulty  2. On the floor |  |  |
| 2. Boiling/hot dishes/ water | 3 | 0. Absent/not accessible  1. May fall onto child with some difficulty  2. Can easily fall onto child |  |  |
| 3. Hot box/ iron box | 2 | 0. Absent/not accessible  1. Maybe reached by child with some difficulty  2. Easily reached by child |  |  |
| 4. Matches | 1 | 0. Absent/not accessible  1. Maybe reached by child with some difficulty  2. Easily reached by child |  |  |
| 5. Corrosives | 4 | 0. Absent/not accessible  1. Maybe reached by child with some difficulty  2. Within easy reach of a child |  |  |

## 3. Electrocution and electric burn hazards

| **Hazard** | **Base score** | **Modifying factor** | **Factor score** | **End score** |
| --- | --- | --- | --- | --- |
| 1. Electric socket | 3 | 0. Absent/inaccessible to child  1. Accessible but child proofed  2. Accessible and not child proofed |  |  |
| 2. Exposed wires of common appliances | 2 | 0. Absent/not accessible  1. Maybe reached by child with some difficulty  2. Within easy reach of a child |  |  |
| 3. Low hanging high tension wire | 4 | 0. Absent/not accessible  1. Maybe reached by child with some difficulty  2. Within easy reach of a child |  |  |
| 4. Immersion rod for heating water | 2 | 0. Absent/not accessible  1. Maybe reached by child with some difficulty  2. Within easy reach of a child |  |  |

## 4. Drowning hazards

| **hazard** | **Base score** | **Modifying factor** | **Factor score** | **End score** |
| --- | --- | --- | --- | --- |
| 1. Large drain | 2 | 0. Absent/not accessible  1. Child may fall in with some difficulty  2. Child will easily fall in |  |  |
| 2. Large open vessels | 2 | 0. Absent/not accessible  1. Child may fall in with some difficulty  2. Child will easily fall in |  |  |
| 3. Coverless manholes /underground water store | 4 | 0. Absent/not accessible  1. Child may fall in with some difficulty  2. Child will easily fall in |  |  |
| 4. Wells | 3 | 0. Absent/not accessible  1. Child may fall in with some difficulty  2. Child will easily fall in |  |  |

## 5. Poisoning hazards

| **Hazard** | **Base score** | **Modifying factor** | **Factor score** | **End score** |
| --- | --- | --- | --- | --- |
| 1. Insecticide/pesticide | 4 | 0. Absent/not accessible  1. Maybe reached by child with some difficulty  2. Within easy reach of a child |  |  |
| 2. Disinfectant/phenyl | 3 | 0. Absent/not accessible  1. Maybe reached by child with some difficulty  2. Within easy reach of a child |  |  |
| 3. Cooking fuel | 2 | 0. Absent/not accessible  1. Maybe reached by child with some difficulty  2. Within easy reach of a child |  |  |
| 4. Paint | 1 | 0. Absent/not accessible  1. Maybe reached by child with some difficulty  2. Within easy reach of a child |  |  |
| 6. Medications | 2 | 0. Absent/not accessible  1. Maybe reached by child with some difficulty  2. Within easy reach of a child |  |  |

## 6. Road traffic injuries

This hazard table to be filled in based on location type (1 to 4 as stated in the beginning of the form)

| **Hazard** | **Base score** | **Modifying factor** | **Factor score** | **End score** |
| --- | --- | --- | --- | --- |
| 1. Entrance of house | 2 | 0. Into a courtyard with a childproof gate  1. Directly onto a by lane  2. Directly onto a street with vehicular traffic |  |  |
| 2. Play area | 3 | 0. Within a designated traffic free zone – courtyard, park etc.  1. Minor roads with occasional light traffic  2. Major thoroughfare with heavy traffic |  |  |
| 3. School | 2 | 0 school gate closed during hours/ crossing guard at opening and closing times  1. Some chance of child running onto road during school  2. No gate/ gate always open and unattended/ children play on road during school |  |  |
| 4. Other locations | 2 | 0. Factor 0 of all three previous rta hazards  1. Factor 1 on any of the three previous rta hazards  2. Factor 2 of any of the three previous rta hazards |  |  |

## 7. Object related mechanical hazards

| **Hazard** | **Base score** | **Modifying factor** | **Factor score** | **End score** |
| --- | --- | --- | --- | --- |
| 1. Heavy objects that may fall on child | 3 | 0. Absent/not accessible  1. May fall onto child with some difficulty  2. Easily will fall on child |  |  |
| 2. Machinery that child may put limb/appendage into – ex. Fans, grinders, heavy machinery | 3 | 0. Absent/not accessible/childproof  1. Child might reach with some difficulty  2. Within easy reach of child |  |  |
| 3. Sharp objects – ex. Knives, tools, etc. | 2 | 0. Absent/not accessible  1. Child may obtain these with some difficulty  2. Child can easily access these |  |  |
| 4. Swallowable objects | 2 | 0. Absent/not accessible  1. Child may obtain these with some difficulty  2. Child can easily access these |  |  |

**8. Calculation of the final environmental hazard risk score**

| Study children | Child home (A) | Play area surrounding home (B) | School  (C) | Baby sitter/other care givers home (D) | Total hazard score  (E= A+B+C+D) | Final environmental hazard risk score for each child  (E/no. of places at which data was collected) |
| --- | --- | --- | --- | --- | --- | --- |
| Child 1 |  |  |  |  |  |  |
| Child 2 |  |  |  |  |  |  |
| Child 3 |  |  |  |  |  |  |
| Child 4 |  |  |  |  |  |  |
